# Recent advances in managing and understanding pyoderma gangrenosum

**DOI:** 10.12688/f1000research.19909.1

**Published:** 2019-12-12

**Authors:** Josh Fletcher, Raed Alhusayen, Afsaneh Alavi

**Affiliations:** 1 Department of Medicine, University of Toronto, Toronto, Ontario, M5S 1A8, Canada; 2 Division of Dermatology, Department of Medicine, Sunnybrook Health Sciences Centre, Toronto, Ontario, M4N 3M5, Canada; 3 Division of Dermatology, Department of Medicine, Women's College Hospital, Toronto, Ontario, M5S 1B2, Canada

**Keywords:** Pyoderma Gangrenosum

## Abstract

Pyoderma Gangrenosum (PG) is a rare neutrophilic dermatosis with multiple different clinical presentations and associated comorbidities. PG has historically been a challenging disorder to diagnose, leading to the development of new diagnostic criteria rather than the traditional approach of a diagnosis of exclusion. The pathophysiology is thought to involve both innate and adaptive immune system dysregulation, neutrophilic abnormalities, environmental, and genetic factors. As of today, no gold standard therapy exists for the treatment of PG, and the literature is restricted to mainly case reports, case series, and 2 small randomized clinical trials. Topical, systemic, and biologic therapy, as well as adequate analgesia and proper wound care all play a role in the management of PG. Recent studies have identified additional cytokines and signalling cascades thought to be involved in the pathogenesis of PG, ultimately leading to the development of new targeted therapies. This review will focus on recent advances in the pathophysiology, clinical presentation and associated comorbidities, diagnosis, and management of PG.

## Introduction

Pyoderma gangrenosum (PG) is a rare auto-inflammatory ulcerative dermatosis with an overall incidence of 5.8 per 100,000 individuals and an increased mortality rate when compared with the general population ^1, 2^. However, given the lack of gold standard for diagnosis, the exact prevalence has yet to be elucidated since PG is commonly under- and over-diagnosed. PG is classified as a neutrophilic dermatosis because of a predominant neutrophilic inflammatory infiltrate in the lesions ^3^. The pathophysiology of PG is not completely understood and is thought to be multifactorial, involving both the innate and adaptive immune system and having a genetic influence ^4, 5^.

There is no widely accepted “gold standard” treatment in the management of PG. Proper analgesia, wound care, and compression therapy are all important tenets in the management of PG. Both topical and systemic therapy can be used, and choice of therapy is dependent on numerous factors, including the number of lesions, size, location, comorbid systemic disease, side effects of the medications, and patient preferences ^4^.

This review will summarize new advances in the pathophysiology, diagnosis, and management of PG in order to assist physicians in better understanding and managing PG and ultimately improving patient care.

## Pathophysiology

The pathophysiology of PG is poorly understood and is thought to involve adaptive and innate immune system dysregulation, neutrophilic abnormalities (chemotaxis, adhesion, and trafficking), abnormal phagocytosis, and genetics ^4^.

**Figure 1.**
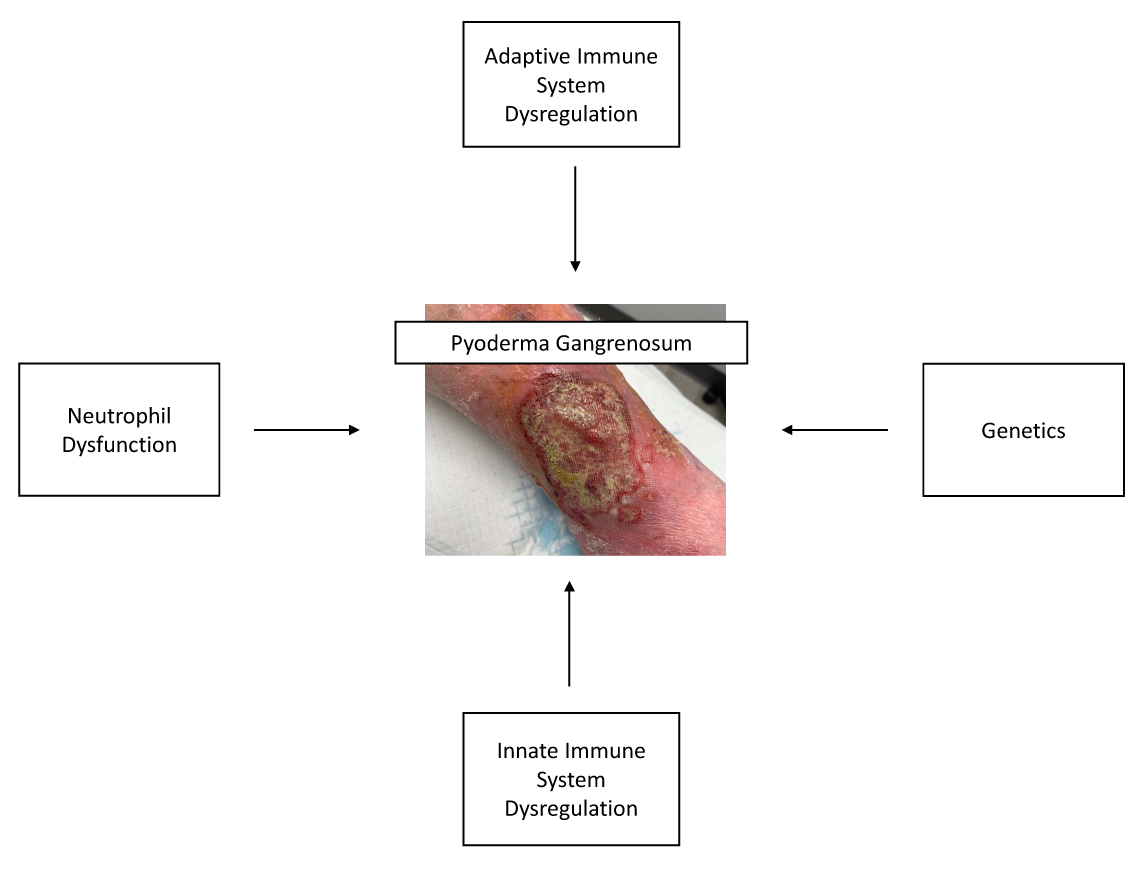
Pathophysiology of pyoderma gangrenosum.

There is increasing evidence in the literature that supports an immunologic etiology for PG. In addition, genetic disorders that alter the immune system are associated with PG ^6^ and multiple novel therapeutic targets have been explored. These are described in more detail below ( Figure 1).

### Neutrophil dysfunction

The abundance of neutrophils in the histopathology of PG has been reported in typical untreated lesions. Histologic analysis of PG lesions demonstrates dermal edema, neutrophilic abscesses, and suppurative inflammation in the dermis that can reach the underlying subcutaneous fat ^6^. This is also evident in PG’s association with other disorders related to neutrophilic dysfunction, such as inflammatory bowel disease (IBD), rheumatoid arthritis (RA), seronegative arthritis, hematologic disorders, and malignancies such as acute myeloid leukemia (AML) ^4, 7^. Interleukin-6 (IL-6), a pro-inflammatory cytokine that plays a role in activation and accumulation of neutrophils, has been found to be elevated in PG lesions ^8^. Interestingly, some studies have shown PG lesions to be related to defects in adhesion and function of neutrophils, suggesting a multifactorial pathogenesis ^7^.

### The role of genetics

Genetics play a role in the pathogenesis of PG, best exemplified by the PG-associated genetic syndromes. The specific mutations that give rise to PG-associated genetic syndromes are all associated with a pro-inflammatory state. Such syndromes and their associated gene mutations are listed in Table 1 ^4^. For example, PAPA (pyogenic arthritis, PG, and acne) syndrome presents as sterile arthritis in childhood, severe cystic acne, pathergy, and recurrent ulcerations. The underlying mutation in PAPA syndrome leads to uncontrolled production of IL-1, thus leading to auto-inflammation ^9^.

**Table 1.** Pyoderma gangrenosum (PG)-associated genetic syndromes and their specific gene mutations ^4^.

Acronym	PG-associated syndrome	Gene mutation
PAPA	Pyogenic arthritis, PG, acne	*PSTPIP1*
PASH	PG, acne, suppurative hidradenitis	*PSTPIP1, NCSTN*
PASS	PG, acne conglobata, suppurative hidradenitis, seropositive spondyloarthropathies	N/A
PAPASH	Pyogenic arthritis, PG, acne, suppurative hidradenitis	*PSTPIP1*
PsAPASH	Psoriatic arthritis, PG, acne, suppurative hidradenitis	N/A

N/A, not available; NCSTN, codes for nicastrin, a protein essential for chemical signaling pathways and for normal immune system functioning; PSTPIP1, proline-serine-threonine phosphatase-interacting protein 1.

Methylene tetrahydrofolate reductase (MTHFR) is an enzyme that assists in the conversion of homocysteine to methionine. Mutations lead to increased levels of homocysteine, resulting in a pro-inflammatory state. Enzymatic co-factors include folic acid, vitamin B _6_, and vitamin B _12_. There are cases of PG associated with this mutation in the literature, improved with vitamin B treatment ^9^.

There are some familial cases of PG in the literature. Three case reports in the pediatric population describe familial PG in both the setting and absence of systemic disease ^10– 12^. Other case reports describe the development of PG in family members after abdominal surgery and trauma ^13^. In addition, there are familial cases of PG-associated genetic syndromes in the literature ^14^. These case reports support the role of genetics in the pathogenesis of PG.

### The innate immune system

Current research is continuing to identify new—and confirm already-known—cytokines and signaling cascades involved in the pathogenesis of PG. The innate immune system signaling pathways, pattern recognition receptor (PRR) pathways (which are associated with autoimmune diseases such as IBD and RA), and Janus kinase (JAK) 1–3 and signal transducer and activator of transcription (STAT) pathways were upregulated in lesional skin compared with non-lesional skin in patients with PG ^15, 16^. In comparisons of lesional skin of individuals with classic ulcerative PG and PG, acne, and suppurative hidradenitis (PASH) syndrome, both showed overexpression of IL-1β, tumor necrosis factor alpha (TNFα), IL-17, endothelial- and leukocyte-selectin, and chemokines IL-8, CXCL16, and RANTES ^17, 18^. Matrix metalloproteinases, a group of enzymes involved in tissue damage, were overexpressed as well ^7^. Biopsies of pre-lesional PG (papules that eventually ulcerated) showed CD3 ^+^ infiltrates and increased inflammatory cytokines, whereas PG lesions showed significant overexpression of IL-1α, IL-1β, IL-6, IL-8, and IL-36α ^19, 20^.

### The adaptive immune system

The adaptive immune system is also thought to play a role in PG given that medications interfering with T-cell function and promoting apoptosis improve symptoms ^7^. The ratio between T regulatory cells and T helper 17 (Th-17) cells was found to be reduced in PG lesions but not in sweet syndrome (which is associated with a milder skin inflammation). T regulatory cells are involved in preventing auto-inflammation and therefore an impairment in the level of these cells may allow the uncontrolled activation of other pro-inflammatory cells and cytokines, such as Th-17 and IL-17 ^21^. In addition, T-cell clonal expansions have been seen in patients with PG early in the disease course ^6^. Histologically, the “cigarette paper” appearance of PG scars was not consistent with a thinning of the dermis, but rather significantly fewer T helper cells and fibroblasts ^20^.

The relationship between external triggers (that is, pathergy) and genetic factors is poorly understood ^5^. Pathergy has been suggested to be caused by increased activity of polymorphonuclear cells because of the presence of neutrophils in pathergic lesions of Behçet disease ^7^. However, further research is needed to confirm this theory in PG.

Wang *et al*. ^20^ (2017) hypothesized that abnormal cytokine expression and an adaptive immune response targeted at pilosebaceous units are responsible for the development of PG. In contrast with psoriasis and venous stasis, ulcerations in PG never occurred at sites of previous ulcers; biopsies of healed and non-lesional skin resulted in ulceration (pathergy) only in the latter. Wang *et al*. hypothesize that this is due to the loss of an autoantigen target, specifically follicular adnexal structures. To further this theory, PG ulcerations (unlike sweet syndrome, scleroderma, and other autoimmune disorders) do not occur in areas lacking follicular adnexal structures, such as the nipple-areolar complex, palmar surface of the hand, and plantar surface of the foot ^20^. Again, future studies are required to test this hypothesis.

## Clinical presentation and comorbidities

There are multiple subtypes of PG, including ulcerative, bullous, pustular, vegetative, and peristomal and post-surgical PG, the latter two being the most commonly pathergic variants. Ulcerative (classic) PG is the most common subtype and usually is seen on the legs. Subtypes can vary in aggression and are associated with different systemic diseases ( Table 2). The majority of individuals have one to three lesions, and lesions typically cover less than 5% of the total body surface area. Lesions heal with a “cigarette paper–like” or cribriform scar, and epithelium from the borders extends into the ulcer (referred to as Gulliver’s sign) ^4, 6^. Given the association between PG and IBD, RA, and hematologic conditions, other extra-cutaneous manifestations may also be present. This includes ocular (scleritis and ulceration), pulmonary (aseptic nodules), splenic, and musculoskeletal (sterile polyarthritis and neutrophilic myositis) involvement ^4^.

**Table 2.** Different clinical presentations of pyoderma gangrenosum and their associated systemic diseases ^3, 4, 22, 23^.

Variant of pyoderma gangrenosum	Common location	Presentation	Associated disease
Ulcerative (classic)	Lower extremities	Rapid progression Violaceous undermined border Very painful	Inflammatory bowel disease (IBD) Arthritis Myeloproliferative disease
Bullous	Face	Superficial bulla Blue-gray border	Myeloproliferative disease (that is, acute myeloid leukemia)
Pustular	Legs Upper trunk	Painful pustules Red halo	IBD
Vegetative	Trunk	Superficial ulcer No violaceous border	None
Peristomal	Near stoma site	Painful ulcer Violaceous undermined border	IBD Enteric malignancies
Post-surgical (procedural) (after nipple piercing)	Surgery site (breast, abdomen most common)	Rapid progression Active and undermined border Pain out of proportion to lesion	Fewer cases of underlying systemic disease (compared with classic form) ^23^
Pyostomatitis vegetans	Buccal gingiva, labial and buccal mucosa	Multiple small white or yellow pustules Erythema Edema	IBD

The average time between surgery and the first symptoms of postoperative PG was 11 days. More invasive procedures and patients with chronic disease have a higher risk of developing this subtype. In one retrospective analysis, about 15% of individuals had post-surgical recurrence or exacerbation of existing lesions ^24^. PG was often diagnosed as a wound infection at first, leading to debridement and ultimately worsening the lesion because of pathergy. Earlier diagnosis and recognition may help prevent morbidity and lessen health-care costs ^22^.

In 2018, a large retrospective review was completed by Ashchyan *et al*., who found that the majority of individuals with PG in the literature were middle-aged white women with an average age at presentation of 51.6 years ^25^. Ulcers were most common on the lower extremities but may also be seen surrounding stomas, in individuals with IBD (peristomal), on the trunk, upper extremities, head or neck, or other locations ^25, 26^. Pathergy was seen in about one third of individuals, was more common over the age of 65, and may be underestimated (if no trauma occurred) ^25, 27^.

Two thirds of individuals have an associated comorbidity and IBD is most common (occurring in 0.5% of patients with IBD), especially in individuals under 65 ^9, 25^. In individuals with IBD and concomitant PG, IBD is diagnosed at a younger age, and they are more likely to have a family history of UC, be of black African origin, have a stoma, and be on immunosuppressive medications. The mean time between diagnosis of IBD and PG was about 6 years, and PG occasionally preceded the diagnosis of IBD ^28^.

Other associated comorbidities include arthritis, malignancy (acute and chronic myeloid leukemia [AML and CML] and non-Hodgkin lymphoma), hematologic disorders (myelodysplastic syndrome, polycythemia vera, IgA monoclonal gammopathy of unknown significance [MGUS], and myelofibrosis), arthritis, hidradenitis suppurativa, and depression ^9, 25, 27^. Hematologic disorders are more common in individuals over 65 years of age ^25^. Three phenotypes of arthritis are seen in association with PG: RA, bowel-associated arthropathy, and progressive erosive seronegative arthritis. PG (bullous PG in particular) can be the initial presentation of leukemia (most commonly AML) and is a poor prognostic sign ^9^. One study found that the mortality rate in patients with leukemia and PG was higher than in patients with non-leukemia hematologic malignancies, but further studies are needed to investigate this relationship ^29^. The paraneoplastic phenomenon can also occur with atypical presentations of PG (atypical locations and vesiculobullous lesions) and is seen more frequently in patients with underlying hematologic disorders ^6^.

Necrotizing neutrophilic dermatosis (NND)—specifically, necrotizing PG (NPG)—is a more severe form of PG seen in critically ill patients with a previous diagnosis of PG. NPG is most commonly associated with hematologic disorders and malignant neoplasms. These patients often mimic necrotizing fasciitis, sepsis, or septic shock given their level of systemic inflammation. Common features of NND include erythematous and necrotizing violaceous margins, ulcerations, pathergy, elevated inflammatory markers, and neutrophil invasion. Tissue biopsy and cultures are critical in distinguishing NPG from necrotizing fasciitis, as NPG does not respond to antibiotics but rather immunosuppressive therapy (that is, steroids) ^30^.

## Diagnosis

Historically, the diagnosis of PG has been challenging given its numerous presentations, clinical similarities with other dermatoses (and other neutrophilic dermatoses), and various associated systemic diseases ^31^. PG therefore has a high misdiagnosis rate: in a study by Weenig *et al*., PG was initially diagnosed and treated in 67% of patients before an alternative diagnosis was made ^32^.

Given the differences in presentation depending on age, Ashchyan *et al*. ^25^ have proposed an age-focused initial evaluation for PG: they use a different approach depending on whether the patient is over or under 65 years old. In their study, diagnosis was based on the criteria proposed by Su *et al*. ^32^ (described in the paragraph and table below),which are centered on PG being a diagnosis of exclusion ^33^. For patients under 65, the history and physical exam should evaluate for the presence of IBD and the threshold for referral to gastroenterology is lower. For patients over 65, physicians should evaluate for hematologic disorders and malignancy with a possible work-up, including a blood smear, monoclonal gammopathy evaluation, and a lower threshold for referral to hematology for bone marrow studies. They suggest that, regardless of age, all patients receive a skin biopsy with tissue culture, age-appropriate malignancy screening, and complete blood count with differential. Depending on the history and physical, an inflammatory arthritis evaluation, autoimmune work-up, and vasculitis screen may be conducted ^25^.

Numerous articles in the literature used the proposed diagnostic criteria for classic ulcerative PG by Su *et al*. ^34^ (2004). The diagnostic criteria, which are based on PG’s being a diagnosis of exclusion, are listed in Table 3. Biopsy is pivotal in the exclusion of other etiologies, and the best location for biopsy is the active ulcer border. The investigation of regular histopathology, including special staining and tissue culture, is required. In order for a diagnosis of PG to be made, both one major criterion and two minor criteria need to be present. Diagnostic criteria for the bullous, pustular, and vegetative variants of PG were also proposed ^34^.

**Table 3.** Comparison of the different diagnostic criteria suggested for pyoderma gangrenosum ^31, 34, 35^.

Proposed diagnostic criteria		
Su *et al*. ^34^ criteria	The Delphi Consensus of International Experts	PARACELSUS score
**Major criteria**		
Other ulcerating conditions excluded (that is, biopsy and other investigations)	Biopsy	Exclude other differential diagnoses
Typical clinical presentation of classic pyoderma gangrenosum ^a^		Reddish-violaceous ulcer border
		Progressive ulceration (developed in <6 weeks)
**Minor criteria ^b^**		
Histopathology findings	Histopathology findings	Histopathology findings
Typical systemic diseases present	Typical systemic diseases present	Typical systemic diseases present inflammatory bowel disease, inflammatory arthritis
Treatment responsive to systemic steroids	Treatment responsive to immunosuppressants	Improvement in symptoms by immunosuppressants
History of pathergy and cribiform scarring	Pathergy	Pathergy
	Cribiform scarring	
	Pain, undermined border, peripheral erythema	Undermined border
		Pain
	Papule, pustule, or vesicle that ulcerated	Irregular ulcer shape
	Multiple ulcerations (at least one on lower leg)	

^a^Progressive (1 to 2 cm/day or increase by 50% in 1 month), painful, irregular and undermined border, violaceous color, preceded by papule, pustule, or bulla. ^b^For the PARACELSUS score, minor criteria have a white background and additional criteria have a gray background.

Given the difficulty in diagnosing PG and excluding other diagnoses, 12 physicians collaborated to create a diagnostic criterion for classic ulcerative PG. The Delphi Consensus of International Experts diagnostic criteria are composed of one major and eight minor criteria ( Table 3). In order for the diagnosis to be made, one major and four minor criteria must be present ^31^. Consensus was not reached regarding the inclusion of hematologic disease as a minor criterion (unlike IBD and inflammatory arthritis). Given the prevalence of hematologic disease in PG, future iterations should consider its addition as a minor criterion. In addition, the use of biopsy as a major criterion may not reflect actual clinical practice ^33^. Interestingly, one study found that only 12% of individuals had histological evidence of a neutrophilic infiltrate and that less than 10% had a biopsy consistent with PG ^27^.

Around the same time as the development of the Delphi criteria above, Jockenhöfer *et al*. ^35^ developed the PARACELSUS score as a separate diagnostic tool for PG. This score was developed on the basis of a review of the literature. The criteria are listed in Table 3. Major criteria (assigned 3 points) were present in more than 95% of individuals, minor criteria (2 points) were present in 61 to 95%, and additional criteria (1 point) were present in not more than 60%. It is worth noting that these criteria were applied to only 60 individuals with lower-extremity PG and 50 patients with venous leg ulcers (control group) and did not consider other locations or ulcerative conditions. Individuals with PG all had a score of more than 10, whereas patients with venous ulcers all had scores of less than 7 ^35^. All three criteria are compared in Table 3, and similar criteria are grouped together.

## Treatment

PG is a challenging condition to manage, and treatment focuses on reducing systemic inflammation. There is a lack of large randomized controlled trials (RCTs) in the literature and therefore the majority of treatment decisions are based on expert opinion, case reports and case series, and small cohort studies. Treatment decisions are personalized to reflect the location, number, and size of lesion(s), extra-cutaneous involvement, underlying systemic disease, side effect profiles, cost, and patient preference ^4^.

### Topical and intralesional therapy

The best evidence in the literature regarding topical therapy is for corticosteroids and tacrolimus. A case series conducted in 2011 found topical therapy to be used most often in peristomal PG, smaller lesions, and localized PG (<5% body surface area, <3 lesions, and lesions <2 cm ^2^) ^5, 36, 37^. One review found that topical tacrolimus 0.3% promoted lesion resolution in mild and localized PG ^37^. Intralesional steroids, applied to the active border of the lesion surrounding the ulcerated area, can also be used in small and localized PG ^4^. However, there is little information on which steroid class to use, the frequency of application, the dosage of tacrolimus to use, and the best dressing to apply afterwards ^36^. Other topical therapies include sodium cromoglycate, nicotine, dapsone, and 5-aminosalicylic acid (5-ASA) ^4^.

Topical therapy was often used alongside other systemic therapies (for example, prednisone), and ulcer size was an important predictive factor in lesion resolution. A prospective cohort study found that less than 50% of individuals had healed (no longer required dressings) with topical therapy alone in a 6-month period and that one third of individuals required systemic therapy ^38^. However, improvements were slow and relapses were not uncommon. Therefore, topical therapy can be used concomitantly with systemic therapy or in patients who are resistant to or cannot tolerate steroids ^37^.

### Systemic therapy

Monotherapy can be used in patients with mild PG. Examples of typical monotherapy agents include steroids, tacrolimus, topical sodium cromoglycate, nicotine, 5-ASA, intralesional triamcinolone, and intralesional cyclosporine. Current research is targeted at the development of new biologic agents that target different inflammatory cytokines and signaling pathways ^6^.

The most commonly used first-line treatment in the management of PG is systemic steroids ^4, 27, 39^. An RCT comparing oral cyclosporin (4 mg/kg per day) with prednisolone (0.75 mg/kg per day) found no difference between these medications in lesion-healing speed (in a 6-week period), treatment response, resolution of wounds, pain, quality of life, treatment failure, and recurrence. Overall, about half of all PG ulcers had healed by 6 months. Therefore, treatment is guided by the side effect profiles of these two medications (serious infections in steroids and hypertension and renal dysfunction in the ciclosporin group) ^39^.

Other systemic therapies (cyclophosphamide, methotrexate, mycophenolate mofetil, sulfasalazine, and azathioprine) have been used in the literature, but more data are needed to evaluate their efficacy in treating PG ^4^. The addition of topical or systemic antibiotics or anti-neutrophilic agents (dapsone and colchicine) has traditionally been based on the provider’s preference. The benefits of using anti-neutrophilic agents are for both their anti-inflammatory effects and prophylaxis against *Pneumocystis jiroveci* ^5^.

Combination therapy is often used in the treatment of PG ^27^. There are few studies comparing different combination therapies in the literature. In two observational studies,100% of in-patients achieved either partial or complete healing when given combination therapy with systemic steroids and another immunomodulator (ciclosporin, dapsone, clofazimine, and cyclophosphamide) ^40, 41^. Currently, the use of well-studied combinations of immunomodulators (for example, cyclosporine/tacrolimus, mycophenolate mofetil, and prednisone) is also recommended in PG ^6^.

### Biologics

Multiple different biologics have been proposed for the treatment of PG. Agents targeting TNFα are the best studied, given their ability to treat coexisting IBD (other than etanercept) ^4^. Steroids appear to be less efficacious in treating PG with comorbid IBD when compared with biologics ^42^. However, the use of biologics is not without potential harm. Rare adverse effects of biologics include lymphoma, congestive heart failure, multiple sclerosis, peripheral neuropathy, and anti-DNA antibody formation. Reactivation of tuberculosis has been seen in the use of anti-TNFα therapy ^6^. Patients should be made aware of the risks of therapy before beginning treatment.

***Tumor necrosis factor antagonists.*** No TNFα antagonist has been proven to be more efficacious than others in the treatment of PG. Their use has been associated with a decrease in C-reactive protein (CRP), IL-1, IL-6, and immune cell adhesion markers ^8^.

Infliximab, the only biologic to have an associated RCT, functions by restoring the ability of T regulatory cells to inhibit aberrant cytokine production ^4^. Given this RCT and its rapid onset of effect, infliximab is often preferred in a clinical setting. Thirty patients were randomly assigned to receive either an infusion of infliximab (5 mg/kg) or placebo at week 0 and were reassessed 2 weeks later. If there was no improvement by week 2, everyone was offered open-labelled infliximab at the same dose; 46% of individuals showed clinical improvement with infliximab (compared with 6% with placebo) by 2 weeks, and 69% had improved by week 6 (21% complete resolution). Individuals with lesions of less than 12 weeks’ duration had a higher improvement/remission rate than those with a longer duration ^43^.

Adalimumab is a humanized IgG1 monoclonal antibody with activity against TNFα. The literature surrounding adalimumab is composed of case reports and small case series; in some of these, it was added to or replaced current therapy because of treatment failure. The majority of the literature showed either complete resolution or partial improvement ^8^. However, the sample size was small and evidence is limited ^4^.

Etanercept functions as a decoy receptor for TNFα and has activity against TNFβ. Data are limited to case reports and small case series, the majority of which showed clinical improvement or complete resolution ^8^. However, etanercept is less efficacious than other TNF antagonists in the treatment of coexisting IBD ^4, 6^.

Golimumab, a newer TNFα inhibitor, led to complete ulcer resolution in 24 weeks in a patient who had failed infliximab and adalimumab. Another novel TNFα inhibitor is certolizumab pegol. Future studies are needed to further evaluate the use of Golimumab and Certrolizumab Pegol PG ^8, 44^.

***IL-12 and IL-23 antagonists.*** Ustekinumab blocks the common p40 subunit of IL-12 and IL-23. These two cytokines are important in neutrophil recruitment through their interaction with Th1 and Th17 cells, respectively. Case reports in the literature demonstrate either partial or complete resolution of PG lesions with ustekinumab; however, more studies are needed to confirm efficacy ^6, 8^.

Tildrakizumab and guselkumab are IL-23 antagonists without simultaneous IL-12 antagonism. Future research is needed to assess their efficacy ^8^.

***IL-1 antagonists.*** As mentioned above, some PG-associated genetic syndromes are associated with a mutation in the *PSTPIP1* gene, leading to increased IL-1 production. IL-1 inhibitors, therefore, have the potential of blocking the downstream effects of this mutation, but the evidence is still limited ^4, 8^.

Anakinra is a competitive inhibitor of IL-1 (both subtypes) with a short half-life (4 to 6 hours). Although the majority of case reports demonstrated partial or complete resolution of ulcers, large daily doses were needed ^8^. In comparison with other biologics, anakinra may be less effective in its management of PG ^4^.

Canakinumab is a monoclonal antibody targeted against IL-1β with a longer half-life (about 1 month) ^8^. Five patients with PG (without systemic disease) who had all failed steroids were given canakinumab. Four of the five individuals had clinical improvement in 16 weeks, and three individuals had complete resolution of their lesions in this time period. However, one patient in this study had new-onset rapidly progressive genital ulcers, likely representing PG at a different location ^19^.

Gevokizumab, another monoclonal antibody targeting IL-1β, showed promise in the treatment of PG; however, the rights to this drug were sold in 2016 ^8^.

***IL-6 antagonists.*** Tocilizumab has been successful in treating PG in a patient with RA and interstitial lung disease (ILD), as ILD is a contraindication to TNFα inhibitors ^5^.

***JAK/STAT inhibitors.*** Tofacitinib is an oral JAK 1 and 3 inhibitor that is currently approved for use in RA and ulcerative colitis. Three patients with treatment-resistant PG and both comorbid Crohn’s disease and inflammatory arthritis were given tofacitinib, leading to complete resolution in two patients and symptom improvement in the third by 12 weeks ^45^.

Ruxolitinib, a JAK-2 inhibitor, was used in a case report of a 64-year-old female with polycythemia vera and bilateral lower-leg PG who had failed multiple immunosuppressive regimens, intravenous immunoglobulin (IVIG), and anakinra. Dramatic healing of the PG lesions was seen within 10 weeks, and complete healing of her lesions was seen after 4 years ^46^.

***Intravenous immunoglobulin therapy.*** IVIG has been used as an adjunctive strategy for treatment-refractive PG, most commonly as a combination therapy with systemic steroids. One study found that patients with solitary PG lesions are more responsive to IVIG than those with two or more lesions whereas factors such as ulcer location had no meaningful significance ^47^. The majority of patients showed clinical improvement, and 53% of these individuals had complete resolution. The most common side effects were headache and nausea. However, patients in this study were older and more likely to have comorbidities than other patients in the literature ^48^. The anti-inflammatory activity of IVIG is likely the reason for lesion resolution ^49^.

***Phosphodiesterase 4 inhibitors.*** Phosphodiesterase 4 (PDE4) is an enzyme produced by immune cells, and inhibition helps modulate different pro-inflammatory signaling cascades. Apremilast, an oral PDE4 inhibitor, inhibits multiple cytokines involved in these signaling cascades ^8^. In the literature, there is one case report of resistant vegetative PG with underlying IgA MGUS. Complete healing of one lesion and partial healing of another were seen when apremilast was used concomitantly with oral prednisone ^50^. Further studies are needed to better characterize this treatment.

### Wound care

Treatment of PG is multifaceted and is not limited to pharmacotherapy. Other aspects of treatment include lifestyle modification (smoking cessation, nutrition, exercise, and prevention of hyperglycemia), the avoidance of triggers and trauma (given the high prevalence of pathergy), wound care, analgesia, prevention of superimposed infection, and compression therapy (to minimize edema) ^4^.

Gentle cleaning of the wound, proper use of topical antimicrobial agents (in the setting of critical colonization) if indicated, a moist wound environment, and control of edema are essential in the management of PG. Multiple dressings have been used in the literature for the treatment of PG. Dressing choice depends on the ulcer’s characteristics (drainage, size, location, and so on). Sharp debridement should be avoided in PG given the high rate of pathergy but may be needed depending on the amount of non-viable tissue ^5^. However, about 30% of individuals have undergone debridement by a wound care specialist because of diagnostic uncertainty ^26^. Other wound management modalities include negative pressure wound therapy and hyperbaric oxygen, both of which show promise ^5^.

Compression therapy is used as an adjunct to immunosuppression in the management of PG as it is paramount in reducing any associated edema and promoting wound healing. One report commented on the importance of multimodal therapy and specifically compression therapy, given the lack of wound healing likely due to prednisone-induced edema ^51^.

### Analgesia and multidisciplinary management

Pain control is an important tenet in the management of PG and often involves the use of non-steroidal anti-inflammatory drugs and opioids. Neuropathic medications can also be used if nerve damage is present ^5^. Opioids may be associated with decreased healing in venous ulcers (in addition to their other adverse effects) and therefore alternative strategies for analgesia should be considered first ^52^.

Given the association between PG and underlying systemic disease, referrals to appropriate subspecialties (that is, gastroenterology, rheumatology, hematology, and so on) are required. In addition, mental health support is warranted given the association between PG and major depressive disorder ^27^.

## Conclusions

PG is a rare neutrophilic dermatosis with a complex pathophysiology and difficult diagnosis. It is important to note that owing to the rarity of this condition, there are very few RCTs. The majority of other publications are cohort studies, case reports, and case studies, many of which have a small sample size. However, current research has been promising and continues to provide new potential targets for therapy. Recently, multiple diagnostic criteria have been proposed to improve the accuracy of diagnosis. Although the pathophysiology of PG is still incompletely understood, the discovery of new inflammatory cytokines and signal cascades has led to the development of novel biologic therapy. Long-term data on the use of immunosuppressive medications, biologics, and small-molecule therapy are lacking. Moreover, it is difficult to assess long-term efficacy, adverse events, and remission rates as outcome measures for PG are not widely available. Additional studies are needed to better characterize these medications and subsequently to compare available treatments.

## Ethics

Written informed consent was obtained from the patient in Figure 1 for the use and publication of this image.
